# The Potential for EBV Vaccines to Prevent Multiple Sclerosis

**DOI:** 10.3389/fneur.2022.887794

**Published:** 2022-06-24

**Authors:** Peter A. Maple, Alberto Ascherio, Jeffrey I. Cohen, Gary Cutter, Gavin Giovannoni, Claire Shannon-Lowe, Radu Tanasescu, Bruno Gran

**Affiliations:** ^1^Division of Clinical Neuroscience, Section of Clinical Neurology, University of Nottingham, Nottingham, United Kingdom; ^2^Department of Neurology, Nottingham University Hospitals NHS Trust, Nottingham, United Kingdom; ^3^Department of Epidemiology, Harvard T. H. Chan School of Public Health, Boston, MA, United States; ^4^Channing Laboratory, Department of Medicine, Brigham and Women's Hospital, and Harvard Medical School, Boston, MA, United States; ^5^Laboratory of Infectious Diseases, National Institute of Allergy and Infectious Diseases, National Institutes of Health, Bethesda, MD, United States; ^6^School of Public Health, University of Alabama at Birmingham, Birmingham, AL, United States; ^7^Blizard Institute, Barts and the London School of Medicine and Dentistry, Queen Mary University of London, London, United Kingdom; ^8^Institute of Immunology and Immunotherapy, The University of Birmingham, Birmingham, United Kingdom; ^9^Mental Health and Clinical Neurosciences Academic Unit, School of Medicine, University of Nottingham, Nottingham, United Kingdom

**Keywords:** Epstein-Barr virus (EBV), prophylactic vaccination, epidemiological evidence, vaccine evaluation, multiple sclerosis

## Abstract

There is increasing evidence suggesting that Epstein-Barr virus infection is a causative factor of multiple sclerosis (MS). Epstein-Barr virus (EBV) is a human herpesvirus, Human Gammaherpesvirus 4. EBV infection shows two peaks: firstly, during early childhood and, secondly during the teenage years. Approximately, 90–95% of adults have been infected with EBV and for many this will have been a subclinical event. EBV infection can be associated with significant morbidity and mortality; for example, primary infection in older children or adults is the leading cause of infectious mononucleosis (IM). A disrupted immune response either iatrogenically induced or through genetic defects can result in lymphoproliferative disease. Finally, EBV is oncogenic and is associated with several malignancies. For these reasons, vaccination to prevent the damaging aspects of EBV infection is an attractive intervention. No EBV vaccines have been licensed and the prophylactic vaccine furthest along in clinical trials contains the major virus glycoprotein gp350. In a phase 2 study, the vaccine reduced the rate of IM by 78% but did not prevent EBV infection. An EBV vaccine to prevent IM in adolescence or young adulthood is the most likely population-based vaccine strategy to be tested and adopted. National registry studies will need to be done to track the incidence of MS in EBV-vaccinated and unvaccinated people to see an effect of the vaccine on MS. Assessment of vaccine efficacy with MS being a delayed consequence of EBV infection with the average age of onset being approximately 30 years of age represents multiple challenges.

## Introduction

Epstein-Barr virus (EBV) is a human herpesvirus, *Human Gammaherpesvirus 4* (1) first isolated during the early 1960s (2). Human herpesviruses have a common structure comprising double-stranded DNA contained within an icosahedral nucleocapsid which is surrounded by a tegument and a host cell-derived outer membrane containing virus glycoproteins. The EBV genome is approximately 170 kilobases long and expresses over 80 different proteins (3–5). These include several viral glycoproteins present on the surface of the virus, including gp350, gH, gL, and gp42 which are important for the attachment and fusion of the virus to the cell and are the targets of prophylactic vaccines (6). EBV is adapted to maintain a long-term existence with the human host as a result of highly effective virus mechanisms capable of evading the human immune response (7, 8). Latency (9) is established following the virus expression of several proteins including the EBV nuclear antigens (EBNAs 1, 2, 3, LP) and latent membrane proteins (LMPs). Antibodies to some of these latent proteins (10), especially EBNA1 have been implicated in the pathogenesis of MS (Table 1).

**Table 1:** EBV proteins and associated antibodies commonly used as infection markers and in investigations of associations with multiple sclerosis^a^.

**EBV protein**	**Function**	**Corresponding antibody**
Virus Capsid (VCA)	A lytic phase protein which is a complex comprising major, minor and smallest capsid proteins. Structural proteins which encapsidate the virus genome (11).	VCA IgM is a marker of acute infection. Initial detection of VCA IgG corresponds also with acute infection; however, following seroconversion VCA IgG usually remains detectable for life (10–14).
Early Antigen (EA)	Protein complexes produced early in the lytic cycle. Two forms identified based on characteristic immunofluorescence patterns. Early antigen diffuse (EA-D) and Early antigen restricted (EA-R). Essential for viral DNA polymerase activity.	EA IgG responses vary with different populations limiting its utility as a general marker of acute infection. High levels of EA-R IgG are associated with Burkitt lymphoma and high levels of EA-D IgG with nasopharyngeal carcinoma. Seropositivity in general populations is approximately 20–30% (10, 14).
Nuclear Antigen 1 (EBNA1)	A DNA binding protein, expressed in virus infected cells, that is produced during latency. It is essential for the persistence and replication of the viral genome (5, 7, 9, 11).	EBNA1 IgG is present in convalescent or past infection; however, a few individuals fail to produce this antibody following infection. Higher levels compared to controls are associated with multiple sclerosis (10–14).
Nuclear Antigen 2 (EBNA2)	Forms complex with DNA-binding cellular transcription factors. Activates viral and cellular promoters as heterodimer. Promotes cell proliferation (5, 7, 9, 11).	EBNA2 IgG has a limited role in EBV diagnosis as it appears earlier than EBNA1 IgG and is present in healthy controls. High levels are reported to be strongly associated with an increased risk of development of multiple sclerosis (10, 11, 13).

a *Numbers in parentheses list relevant references*.

EBV infection typically occurs during early childhood and in the great majority of young children it is asymptomatic or subclinical; however, in older children and adults EBV infection is a major cause of infectious mononucleosis (12, 13). The most frequent signs and symptoms of infectious mononucleosis (IM) in young adults are sore throat (95–98%), cervical lymphadenopathy (80–88%), and fatigue (70–78%) of variable duration (median 15 days). The reported rates of presentation of IM following primary EBV infection in young adults are variable e.g. 25–74% (12, 13). Other frequent symptoms of IM include fever, headache, loss of appetite, myalgia and upper respiratory tract symptoms and rarer clinical findings include abdominal pain, hepatomegaly, splenomegaly, nausea, and vomiting (12, 13). In cases when the immune system is compromised, severe EBV-mediated disease may occur such as post-transplant lymphoproliferative disorders following immunosuppression (15). Chronic active EBV infection is a rare lymphoproliferative disease associated with high morbidity and mortality in which an ineffective T-cell response enables the clonal proliferation of infected cells in patients without an apparent immune defect (14). Defective immune responses due to inherited genetic conditions may also result in failure to contain EBV replication such as occurs with X-linked lymphoproliferative syndrome (16). EBV is an oncogenic virus, and it is associated with the development of several lymphomas and carcinomas (17). Finally, EBV infection is associated with the development of several autoimmune diseases (11).

EBV is frequently shed in oral fluid (18) and transfer of the virus via sharing saliva is the main route of virus transmission. EBV can also be transmitted via sexual activity and transplantation (12, 19). Epithelial cells and B-lymphocytes are the primary sites of infection (20). Latency is established in memory B cells and the expression of latent proteins are key for immune avoidance and persistence of the virus within the host (7, 9, 21).

In England and Wales, EBV seroprevalence studies show two peaks of infection in children less than 5 years old (seroprevalence of 35% in 1–4 year-olds) and in young adults (seroprevalence of 72% in 15–19 year-olds) (22). Significant disparities exist with the age of acquisition of infection with a trend toward older children experiencing infection in certain population groups and geographical regions (23). EBV infection occurs worldwide with 90–95% of adults having serological evidence of EBV infection (24).

## Multiple Sclerosis

MS is the most common chronic inflammatory demyelinating disease of the central nervous system (CNS), affecting at least 2.5 million people worldwide. It is one of the most frequent causes of disability in young adults (25).

The etiology of MS remains unknown, however, EBV may be causally related (26). The pathogenesis of MS is thought to be mediated by the immune system. Evidence for immune-mediated mechanisms (27) comes from the pathology of disease (28), the contribution of genes of the immune response to disease susceptibility (29), experimental observations in relevant animal models (30), and from the efficacy of immunotherapies in those affected by MS (31).

MS lesions are characterized by loss of CNS myelin, axonal damage, activated microglia, and inflammatory infiltrates of peripheral immune cells including T and B lymphocytes and plasma cells (32), as well as gliosis in more advanced disease. Both white matter and gray matter myelin are affected in the brain and spinal cord. The pathology of MS in its different stages is reviewed in detail elsewhere (33).

Over 200 gene polymorphisms contribute to MS susceptibility (34). The strongest association is with genes of the immune response, primarily certain class I and II alleles of the human leucocyte antigen / major histocompatibility complex (29). Alleles of the HLA-DRB1 locus confer a higher risk of MS and interact with environmental factors known to influence susceptibility such as smoking and solvent exposure, EBV infection (35), childhood and adolescent obesity (36) and low vitamin D levels (37). Other non-HLA genes of the immune response have a more modest effect (34).

Environmental factors, both infectious and non-infectious, influence the risk of developing MS by interacting with the genetic variation that predisposes to autoimmune responses (38, 39). The incidence and prevalence of MS vary geographically, with the prevalence increasing with geographic latitude. A number of studies have found an inverse relationship between sun exposure, ultraviolet radiation exposure, or serum vitamin D levels, and the prevalence of MS (40). Smoking increases the risk of developing MS and may also be a risk factor for disease progression (41).

Among infectious factors, viral infections have been reported to be associated with MS, particularly when they occur in adolescence. Evidence for the role of EBV infection in the development of MS is compelling to the extent that it is now considered a putative causative agent (see below).

Understanding the factors that set off MS can potentially enable prevention. Lifestyle and environmental factors can be subject to possible interventions, in particular in individuals at risk for developing MS, such as relatives of people with MS. EBV infection is an MS trigger (26) and preventing it could prevent MS.

### Epidemiology of EBV in Relation to MS

A link between EBV infection and MS was first suspected over 40 years ago based on the similarity between the epidemiology of MS and infectious mononucleosis (IM), which is a common manifestation of primary EBV infection occurring during adolescence or later in life (42). Further support for this hypothesis came from the observation that individuals with a history of IM are 2–3-fold more likely to develop MS than individuals with asymptomatic EBV infection (43–45). This association could be explained by confounding by hygiene – a proposition known as the “hygiene hypothesis”: IM is a marker of a high level of hygiene during childhood, which predisposes to MS by priming the immune system toward pro-inflammatory responses (46). Such a hypothesis could be tested by determining the MS risk in young adults who are EBV negative. Since EBV negativity is a reliable marker of a hygienic upbringing, if high levels of hygiene were causally related to MS, these individuals should have an increased MS risk. Paradoxically, the results of several cross-sectional studies have suggested that not only do individuals with high levels of hygiene have a greater risk of MS, but, as long as they remain EBV negative, they have a markedly lower risk than EBV infected individuals (44, 47). Similar results were obtained in pediatric MS (48, 49). These findings, together with evidence from several longitudinal studies that circulating IgG antibody titres to EBNA1 give a robust prediction of future MS risk (50–54), suggest that EBV plays a direct causal role in MS. Alternative explanations, however, remain possible. On one hand, the low risk of MS in EBV negative individuals has been inferred from case-control studies that included individuals recruited several years after MS diagnosis – by design, these studies could not exclude the possibility that EBV infection occurred soon after MS. On the other, it has been argued that higher anti-EBNA1 titres could result from an immune dysregulation preceding the onset of neurological symptoms of MS.

Only recently have these alternative explanations been refuted and compelling evidence has been provided that EBV causes MS. This demonstration relied on the longitudinal investigation of a cohort comprising over 10 million young adults (26). At the time of recruitment, about 5% of these individuals were EBV seronegative, thus providing a unique setting to investigate the temporal relation between EBV infection and MS risk. The results were striking – MS risk remained close to zero in EBV negative individuals but increased 32-fold after infection with EBV. In contrast, no increase in MS risk was found after infection with cytomegalovirus, which, like EBV, is mostly transmitted by saliva. Further, serum levels of neurofilament light chain (NfL), a sensitive, albeit not disease-specific, biomarker of neuroaxonal degeneration, were used to examine the temporal relation between EBV infection and the beginning of the putative pathological process leading to MS. Serum NfL levels increased up to 6 years before the clinical onset of MS and can thus be used as a marker of the time of potential disease initiation (26). Serum NfL levels in individuals who went on to develop MS were similar to those of individuals who remained healthy before and around the time of EBV seroconversion but increased after EBV seroconversion. This finding demonstrates that EBV infection precedes the earliest sign of the probable pathological process leading to MS. In the same investigation, the concern that non-specific immune dysregulation could explain the association between EBV, and MS was further examined by conducting a comprehensive agnostic search of the anti-virome antibody response using VirScan, a phage-based immunoprecipitation and sequencing technology (55). This search, conducted using pre- and post-onset serum samples from 30 MS cases and 30 closely matched controls, revealed that only EBV-derived peptides elicited stronger responses in MS cases than controls.

The biological plausibility (56), temporal sequence, and particularly the strength of the EBV-MS association, which virtually exclude confounding by any known or hypothetical risk factor, support the conclusion that EBV is the leading cause of MS (57).

### Current Status of EBV Vaccines

Two types of EBV vaccines are under development; a prophylactic vaccine to prevent infection or disease, and a therapeutic vaccine to treat persons with EBV-associated cancers (Table 2). The prophylactic vaccine furthest along in clinical trials contains the major viral glycoprotein gp350 (58), which is important for virus attachment to B cells, in alum/monophosphoryl lipid A adjuvant. In a phase 2 study the vaccine reduced the rate of infectious mononucleosis by 78% but did not prevent EBV infection (59). A phase 1 study of an EBV peptide, derived from EBNA3A, in tetanus toxoid and oil and water emulsion, showed a trend in reduction of infectious mononucleosis (60). More recently, several other vaccines have been tested in small animal models and some in non-human primates. These vaccines contain gp350 or other viral glycoproteins including gH/gL and/or gB, which are required for fusion of the virus to B cells and epithelial cells, and gp42, which is essential for the virus to fuse to B cells. Vaccine formats have included display of gp350 or gH/gL/gp42 on ferritin nanoparticles in Sigma Adjuvant System (61, 62), trimeric recombinant gB and gH/gL in alum and CpG (63), Newcastle disease virus-like particles with gp350, gH/gL/gp42 or gB in alum/monophosphoryl lipid A (64), and EBV virus-like particles deleted for certain EBV latent and lytic genes (65). Vaccination of small animals with each of these vaccines induced EBV neutralizing antibodies to B cells and/or epithelial cells, and antibodies elicited by some of these vaccines inhibited EBV-mediated glycoprotein fusion to B cells and epithelial cells. In January 2022, Moderna initiated a clinical trial of an mRNA vaccine containing EBV gp350, gH/gL, and gp42 [clinicaltrials.gov NCT05164094 (66)]. A clinical trial of a gp350 ferritin nanoparticle vaccine in Matrix-M1 began in 2022 at the NIH [clinicaltrails.gov NCT04645147 (67)]. While the ultimate goal of a prophylactic vaccine would be to prevent infection with the virus, the vaccine might not completely block infection but instead, reduce EBV associated diseases including malignancies and autoimmune diseases associated with the virus.

**Table 2:** Epstein-Barr virus prophylactic vaccine clinical trials: past and present^a^.

**Vaccine**	**Manufacturer**	**Clinical trial/publication**	**Outcome**
Recombinant gp350	GlaxoSmithKline Biologicals, Rixensart, Belgium	Phase 1 and phase 2 studies to evaluate safety and immunogenicity of a recombinant gp350 EBV vaccine in healthy adults (58)	Phase 1 (59 subjects evaluated). One severe adverse event. Phase 1 and 2 studies (79 subjects evaluated). One severe adverse event.
Recombinant gp350 and ASO_4_ adjuvant system	GlaxoSmithKline Biologicals, Rixensart, Belgium	Recombinant gp350 vaccine for infectious mononucleosis: a phase 2, randomized, double-blind, placebo-controlled trial to evaluate the safety, immunogenicity, and efficacy of an EBV vaccine in healthy young adults. (59)	A total of 178 EBV seronegative subjects were evaluated. No subject discontinued medication for reasons of safety or reactogenicity. In an intention to treat analysis, IM cases were distributed as follows: 9 cases (1 probable and 8 definite) were found in the placebo group and 2 cases (both definite) were found in the vaccine group (*p* = 0.03; α = 0.05, by 1-sided Fisher's exact test).
CD8+ T-cell synthetic peptide HLA B*0801-restricted epitope and tetanus toxoid vaccine	Queensland Institute of Medical Research, Australia	Phase 1 trial of a CD8+ T-cell peptide epitope-based vaccine for infectious mononucleosis. (60)	A total of 14 subjects were evaluated. No serious adverse events were reported. Trial too small to estimate vaccine efficacy. Vaccine immunogenic in most individuals.
mRNA-1189	Moderna TX, Inc.	A phase 1, randomized, observer-blind, placebo-controlled, dose-ranging study of an EBV candidate vaccine, mRNA-1189, in 18- to 30-year-old healthy adults. Trial ongoing. Estimated completion date June 2023. NCT05164094	Main outcome is to evaluate the safety and reactogenicity of mRNA-1189 in 18- to 30-year-old healthy adults. Secondary outcome is to evaluate vaccine immunogenicity.
EBV gp350-Ferritin nanoparticle vaccine adjuvanted with Matrix M1	National Institute of Allergy and Infectious Diseases, USA	A phase 1 study of the safety and immunogenicity of an EBV gp350-Ferritin nanoparticle vaccine in healthy adults with or without EBV infection. Trial ongoing. Estimated completion date July 2025. NCT04645147	Main outcome is to evaluate the safety and reactogenicity of gp350-Ferritin nanoparticle vaccine in 18- to 29-year-old healthy adults.

a *Numbers in parentheses list relevant references*.

To prevent the development of EBV-associated diseases, vaccines designed to also harness T cell responses to the viral proteins could be employed to generate antiviral T cells alongside the neutralizing antibodies. T cells that target EBV structural proteins that are delivered into the cells as pre-formed virion components could eradicate the newly infected cells before they express growth transforming viral latency proteins; thus, such vaccines might prevent the establishment of a permanent latent infection. Indeed, T cells have been identified that recognize epitopes from structural proteins in newly infected B cells, including gp350, gH, gL and gB (68) as well as tegument and capsid proteins (68, 69).

Importantly, therapeutic vaccine strategies can also be employed to treat individuals with EBV-associated diseases by boosting the existing antiviral T cell responses or even inducing novel antiviral responses. The advantage to such strategies is their exquisite tumor-specificity that allows the cancer to be targeted with minimal risk to normal tissue. In such trials, EBV proteins expressed in latently infected tumor cells such as EBV nuclear antigens (EBNAs) or latent membrane proteins (LMPs) have been used as vaccine targets. The initial therapeutic vaccine trials in nasopharyngeal carcinoma (NPC) patients employed autologous monocyte-derived dendritic cells (DCs) loaded with LMP2 CD8+ T cell epitope peptides. The first trial showed that 9 out of 18 vaccinated patients exhibited an increase in circulating LMP2-specific T cells, of whom 2 had partial clinical responses (70); similarly, the second trial showed increased circulating LMP2-specific T cells in 7 out of 16 vaccinated patients but no clinical responses (71). Importantly, these trials only used a limited number of pre-defined LMP2 CD8+ T cell epitopes, so to expand the range of T cell specificities an alternative approach using autologous DCs transduced with an adenovirus expressing truncated LMP1 and full-length LMP2 was employed. However, patients on this trial had been heavily pre-treated with cytotoxic chemotherapy and no expansions in T cell responses were observed, yet out of 12 patients, one partial clinical response and two instances of stable disease were achieved (72). Since these DC-based vaccination trials were initiated, non-cell based therapeutic vaccines have been developed that employ recombinant viral vectors expressing EBNA1, LMP1 and/or LMP2 and have undergone clinical trials in NPC patients. An initial phase 1 trial using an adenoviral vector expressing LMP2 induced a dose-dependent increase in the number of LMP2-specific CD3+ CD4+ T cells (73). In phase 1 trials of a modified vaccinia Ankara (MVA) virus expressing the carboxyl terminus of EBNA1 fused to full-length LMP2 designed to induce both CD4+ and CD8+ T cell responses respectively, a two-fold increase in the T cell response to one or both of the EBV proteins was observed in NPC patients from Hong Kong (74) and the UK (75). Furthermore, the vaccination boosted a broad range of CD4+ and CD8+ T cell responses against EBNA1 and LMP2. Since portions of EBNA1 have been reported to mimic cellular proteins resulting in cross-reacting antibodies that could affect the nervous system (76), these portions of EBNA1 might be deleted from a vaccine to reduce the risk of inducing an immune response to these cellular proteins. To summarize, while therapeutic vaccines have had modest clinical activity, relatively few studies have been performed and many of the patients had previously received cytotoxic chemotherapy which would likely impair their response to vaccines.

### Potential Benefits of EBV Vaccines in the Prevention of MS

EBV is not only causally linked to MS, but is considered to play a potentially causal role in other autoimmune disease such as systemic lupus erythematosus (SLE) (77) and is associated with several malignancies including nasopharyngeal carcinoma, gastric carcinoma, and Burkitt, Hodgkin, and other lymphomas (78). EBV's oncogenic potential has resulted in an emphasis on developing vaccines that induce sterilizing immunity with the objective of preventing EBV infection (79, 80). However, it is debatable whether sterilizing immunity is necessary for preventing MS. EBV-associated infectious mononucleosis (IM) is a stronger risk factor than asymptomatic EBV infection for MS (43, 81, 82). High titres of anti-EBNA EBV antibodies at least in part represent a risk factor different from IM (83). High antibody titres to EBNA1 are associated with a greater MS risk and may indicate an inability to control EBV viral loads. This may be due to the MS-associated HLA DR15 haplotype, which may be associated with reduced control of EBV. In a humanized mouse model of EBV infection, the MS-associated HLA DR15 haplotype was associated with higher EBV viral loads (84). Therefore, non-sterilizing immunity from a vaccine that protects against IM and reduces immune responses to EBNA1, but not EBV infection, may be sufficient to reduce the incidence of MS and other autoimmune diseases.

## Conclusions

An EBV vaccine to prevent IM in adolescence or young adulthood is the most likely population-based vaccine strategy to be tested and adopted. Based on the recent experience with COVID-19 vaccines the general population is likely to be risk-averse when it comes to the potential uptake of a new EBV vaccine (85). Therefore, it is likely that an EBV vaccine will be targeted at 12–13-year-olds, piggybacking on the HPV vaccine program. Although seroprevalence rates vary, over 50% of children in the general population will have already seroconverted by the age of 5 (86). This is arguably why an EBV vaccine will have to target a younger age group, e.g., 2–3 years of age before children are exposed to much horizontal transfer of EBV. For vertical transmission, i.e., mother-to-child transmission that occurs in lower-income environments, vaccination may have to occur even earlier. This is relevant for using an EBV vaccine to prevent some cancers such as Burkitt lymphoma and nasopharyngeal cancer.

Once a population-based primary EBV vaccination has been adopted, national registry studies will need to be done to track the incidence of MS, other autoimmune disease and EBV-associated lymphomas and cancers in EBV-vaccinated and unvaccinated people to see a delayed effect of the vaccine. With MS being a delayed consequence of EBV infection with the average age of onset being approximately 30 years of age this study will take a decade or more to deliver an answer.

In high-prevalence countries where MS has an annual incidence of 7.5 per 100,000 person-years, for a vaccine to reduce the incidence by two-thirds, preliminary estimates are that over 500,000 subjects would be needed (87). A registry of vaccinated individuals could be used for a matched cohort study, in which the exposure is EBV vaccination, and the outcome is MS. A two-sided test of whether the hazard ratio is one with an overall sample size of 512,138 subjects (of which 256,069 are in the control group and 256,069 are in the treatment group) achieves 80% power at a 0.05 significance level when the hazard ratio is actually 0.33 (a 67% reduction). The number of events required to achieve this power is 25.5. It is anticipated that proportions of subjects having the event during the study is 0.000075 for the control group and 0.00002475 for the vaccine group (rate 1/3 of the 7.5 per 100,000 incidence). These results assume that the hazard ratio is constant throughout the study and that Cox proportional hazards regression is used to analyze the data.

Targeting EBV-negative older people at high risk of getting MS would potentially be quicker. However, making it through the early lifespan and not being infected may enrich for a selection bias that reduces the incidence. By being older the lag time to expected to MS development is shorter. While the duration of the study might be shortened, the sample size to establish the reduced incidence would remain essentially at 500,000 and since 80 to 95% of individuals are EBV-positive by age 20, the amount of screening necessary would involve over 3 million subjects to obtain the eligible randomized cohort assuming 100% consent to enroll. One proposal is to recruit first and second-degree relatives of people who have MS as these people will be more likely to volunteer for the study. As with the general population study above, the majority of older relatives >16 years of age would have already been infected with EBV and those destined to get MS may already have subclinical disease. However, just as older and immunosuppressed people benefit from the VZV vaccine in preventing shingles, EBV-seropositive people may benefit from the EBV vaccine as well.

Immunological data suggest that people with MS have a problem controlling EBV and have elevated antibody titres against EBNA1 (52). They have more EBNA1 reactive CD4+ T-cells (88), which respond to a larger repertoire of epitopes distributed across the EBNA1 protein (88). In comparison, T-cells from healthy controls only react to the immunodominant portion of the protein (89). It has also been shown that the poor control of EBV in persons with MS is due to cytotoxic CD8+ T-cells being exhausted and poorly responsive to EBV (90, 91). This is the rationale for the use of autologous and HLA-restricted allogeneic CTLs as a treatment for MS (92, 93). It is, therefore, possible that an EBV vaccine may stimulate immunity to overcome this T-cell exhaustion and reduce the chances of someone developing MS by improved control of EBV. Therefore, there is a strong argument to vaccinate all-comers. In reality, a study of older EBV seronegative high-risk individuals is not feasible. Based on vaccinating all-comers with an assumption that the familial incidence of MS is 300 per 100,000, for 90% power and a 3-fold reduction in the incidence from 300 to 100 per 100,000 a sample size of about 43,000 is required.

In summary, the more pragmatic approach would be doing a matched case-control study using a registry of EBV vaccinated individuals with the outcome being MS and in parallel an all-comer trial, agnostic of EBV status, in high-risk first- and second-degree family members using first clinical event compatible with demyelination as the primary outcome.

## Data Availability Statement

The original contributions presented in the study are included in the article/supplementary material, further inquiries can be directed to the corresponding author/s.

## Author Contributions

PM, AA, JC, GG, CS-L, and RT wrote sections of the manuscript and reviewed it. GC reviewed the manuscript and performed several statistical calculations. BG planned and organized the manuscript and contributed to the writing of the manuscript and reviewed it. All authors contributed to the article and approved the submitted version.

## Funding

JC is supported by the intramural research program of the National Institute of Allergy and Infectious Diseases. BG and PM are supported by the Italian MS Society (FISM grant 2020/R-Multi/050). RT is supported by the UK MRC (CARP MR/T024402/1) and FISM.

## Conflict of Interest

The authors declare that the research was conducted in the absence of any commercial or financial relationships that could be construed as a potential conflict of interest.

## Publisher's Note

All claims expressed in this article are solely those of the authors and do not necessarily represent those of their affiliated organizations, or those of the publisher, the editors and the reviewers. Any product that may be evaluated in this article, or claim that may be made by its manufacturer, is not guaranteed or endorsed by the publisher.

## References

[1] Walker PJ Siddell SG Lefkowitz EJ Mushegian AR Adriaenssens EM Alfenas-Zerbini P. Changes to virus taxonomy and to the international code of virus classification and nomenclature ratified by the international committee on taxonomy of viruses (2021). Arch Virol. (2021) 166:2633–48. 10.1007/s00705-021-05156-1 34231026

[2] Epstein MA Achong BG Barr YM. Virus particles in cultured lymphoblasts from burkitt's lymphoma. Lancet. (1964) 1:702–3. 10.1016/S0140-6736(64)91524-7 14107961

[3] Baer R Bankier AT Biggin MD Deininger PL Farrell PJ Gibson TJ. DNA sequence and expression of the B95-8 Epstein-Barr virus genome. Nature. (1984) 310:207–11. 10.1038/310207a0 6087149

[4] Dolan A Addison C Gatherer D Davison AJ Mcgeoch DJ. The genome of Epstein-Barr virus type 2 strain AG876. Virology. (2006) 350:164–70. 10.1016/j.virol.2006.01.015 16490228

[5] Young LS Yap LF Murray PG. Epstein-Barr virus: more than 50 years old and still providing surprises. Nat Rev Cancer. (2016) 16:789–802. 10.1038/nrc.2016.92 27687982

[6] Balfour HHJr Schmeling DO Grimm-Geris JM. The promise of a prophylactic Epstein-Barr virus vaccine. Pediatr Res. (2020) 87:345–52. 10.1038/s41390-019-0591-5 31641280 PMC8938943

[7] Cohen JI. The biology of Epstein-Barr virus: lessons learned from the virus and the host. Curr Opin Immunol. (1999) 11:365–70. 10.1016/S0952-7915(99)80062-4 10448133

[8] Murata T Sugimoto A Inagaki T Yanagi Y Watanabe T Sato Y. Molecular basis of epstein-barr virus latency establishment and lytic reactivation. Viruses. (2021) 13:2344. 10.3390/v13122344 34960613 PMC8706188

[9] Young LS Dawson CW Eliopoulos AG. The expression and function of Epstein-Barr virus encoded latent genes. Mol Pathol. (2000) 53:238–47. 10.1136/mp.53.5.238 11091847 PMC1186976

[10] Almohmeed YH Avenell A Aucott L Vickers MA. Systematic review and meta-analysis of the sero-epidemiological association between Epstein Barr virus and multiple sclerosis. PLoS ONE. (2013) 8:e61110. 10.1371/journal.pone.0061110 23585874 PMC3621759

[11] Houen G Trier NH. Epstein-Barr virus and systemic autoimmune diseases. Front Immunol. (2021) 11:587380. 10.3389/fimmu.2020.587380 33488588 PMC7817975

[12] Dunmire SK Verghese PS Balfour HHJr. Primary Epstein-Barr virus infection. J Clin Virol. (2018) 102:84–92. 10.1016/j.jcv.2018.03.001 29525635

[13] Balfour HHJr Dunmire SK Hogquist KA. Infectious mononucleosis. Clin Transl Immunol. (2015) 4:e33. 10.1038/cti.2015.1 25774295 PMC4346501

[14] Odumade OA Hogquist KA Balfour HHJr. Progress and problems in understanding and managing primary Epstein-Barr virus infections. Clin Microbiol Rev. (2011) 24:193–209. 10.1128/CMR.00044-10 21233512 PMC3021204

[15] Lindsay J Othman J Heldman MR Slavin MA. Epstein-Barr virus posttransplant lymphoproliferative disorder: update on management and outcomes. Curr Opin Infect Dis. (2021) 34:635–45. 10.1097/QCO.0000000000000787 34751183 PMC8589110

[16] Rezaei N Mahmoudi E Aghamohammadi A Das R Nichols KE. X-linked lymphoproliferative syndrome: a genetic condition typified by the triad of infection, immunodeficiency and lymphoma. Br J Haematol. (2011) 152:13–30. 10.1111/j.1365-2141.2010.08442.x 21083659

[17] Farrell PJ. Epstein-Barr Virus and Cancer. Annu Rev Pathol. (2019) 14:29–53. 10.1146/annurev-pathmechdis-012418-013023 30125149

[18] Johnson KH Webb CH Schmeling DO Brundage RC Balfour HHJr. Epstein-Barr virus dynamics in asymptomatic immunocompetent adults: an intensive 6-month study. Clin Transl Immunol. (2016) 5:e81. 10.1038/cti.2016.28 27350880 PMC4910122

[19] Higgins CD Swerdlow AJ Macsween KF Harrison N Williams H Mcaulay K. A study of risk factors for acquisition of Epstein-Barr virus and its subtypes. J Infect Dis. (2007) 195:474–82. 10.1086/510854 17230406

[20] Chen J Longnecker R. Epithelial cell infection by Epstein-Barr virus. FEMS Microbiol Rev. (2019) 43:674–83. 10.1093/femsre/fuz023 31584659 PMC7317989

[21] Guo R Gewurz BE. Epigenetic control of the Epstein-Barr lifecycle. Curr Opin Virol. (2021) 52:78–88. 10.1016/j.coviro.2021.11.013 34891084 PMC9112224

[22] Morris MC Edmunds WJ Hesketh LM Vyse AJ Miller E Morgan-Capner P. Sero-epidemiological patterns of Epstein-Barr and herpes simplex (HSV-1 and HSV-2) viruses in England and Wales. J Med Virol. (2002) 67:522–7. 10.1002/jmv.10132 12115998

[23] Dowd JB Palermo T Brite J McDade TW Aiello A. Seroprevalence of Epstein-Barr virus infection in U.S. children ages 6-19, 2003-2010. PLoS ONE. (2013) 8:e64921. 10.1371/journal.pone.0064921 23717674 PMC3661547

[24] Winter JR Jackson C Lewis JE Taylor GS Thomas OG Stagg HR. Predictors of Epstein-Barr virus serostatus and implications for vaccine policy: A systematic review of the literature. J Glob Health. (2020) 10:010404. 10.7189/jogh.10.010404 32257152 PMC7125428

[25] Dobson R Giovannoni G. Multiple sclerosis - a review. Eur J Neurol. (2019) 26:27–40. 10.1111/ene.13819 30300457

[26] Bjornevik K Cortese M Healy BC Kuhle J Mina MJ Leng Y. Longitudinal analysis reveals high prevalence of Epstein-Barr virus associated with multiple sclerosis. Science. (2022) 375:296–301. 10.1126/science.abj8222 35025605

[27] Murua SF Farez MF Quintana FJ. The immune response in multiple sclerosis. Annu Rev Pathol. (2022) 17:121–39. 10.1146/annurev-pathol-052920-040318 34606377

[28] Tobin WO Kalinowska-Lyszczarz A Weigand SD Guo Y Tosakulwong N Parisi JE. Clinical correlation of multiple sclerosis immunopathologic subtypes. Neurology. (2021) 97:e1906–13. 10.1212/WNL.0000000000012782 34504026 PMC8601208

[29] Kim W Patsopoulos NA. Genetics and functional genomics of multiple sclerosis. Semin Immunopathol. (2022) 44:63–79. 10.1007/s00281-021-00907-3 35022889

[30] T Hart B. A. Kap Y.S. (2017). An essential role of virus-infected B cells in the marmoset experimental autoimmune encephalomyelitis model. Mult Scler J Exp Transl Clin. 3:2055217317690184. 10.1177/2055217317690184 28607749 PMC5466146

[31] Martin R Sospedra M Rosito M Engelhardt B. Current multiple sclerosis treatments have improved our understanding of MS autoimmune pathogenesis. Eur J Immunol. (2016) 46:2078–90. 10.1002/eji.201646485 27467894

[32] Machado-Santos J Saji E Troscher AR Paunovic M Liblau R Gabriely G. The compartmentalized inflammatory response in the multiple sclerosis brain is composed of tissue-resident CD8+ T lymphocytes and B cells. Brain. (2018) 141:2066–82. 10.1093/brain/awy151 29873694 PMC6022681

[33] Lassmann H. Multiple Sclerosis Pathology. Cold Spring Harb Perspect Med. (2018) 8a028936. 10.1101/cshperspect.a028936 29358320 PMC5830904

[34] Baranzini SE Oksenberg JR. The genetics of multiple sclerosis: from 0 to 200 in 50 years. Trends Genet. (2017) 33:960–70. 10.1016/j.tig.2017.09.004 28987266 PMC5701819

[35] Munz C. Kissing genetic MS risk loci to life. EBioMed. (2021) 72:103594. 10.1016/j.ebiom.2021.103594 34563927 PMC8477131

[36] Hedstrom AK Hillert J Brenner N Butt J Waterboer T Strid P. DRB1-environment interactions in multiple sclerosis etiology: results from two Swedish case-control studies. J Neurol Neurosurg Psychiatry. (2021) 92:717–22. 10.1136/jnnp-2020-325676 33687974 PMC8223646

[37] Cocco E Meloni A Murru MR Corongiu D Tranquilli S Fadda E. Vitamin D responsive elements within the HLA-DRB1 promoter region in Sardinian multiple sclerosis associated alleles. PLoS ONE. (2012) 7:e41678. 10.1371/journal.pone.0041678 22848563 PMC3404969

[38] Morandi E Tanasescu R Tarlinton RE Constantinescu CS Zhang W Tench C. The association between human endogenous retroviruses and multiple sclerosis: a systematic review and meta-analysis. PLoS ONE. (2017) 12:e0172415. 10.1371/journal.pone.0172415 28207850 PMC5313176

[39] Reich DS Lucchinetti CF Calabresi PA. Multiple Sclerosis. N Engl J Med. (2018) 378:169–80. 10.1056/NEJMra1401483 29320652 PMC6942519

[40] Zarghami A Li Y Claflin SB Van Der Mei I Taylor BV. Role of environmental factors in multiple sclerosis. Expert Rev Neurother. (2021) 21:1389–408. 10.1080/14737175.2021.1978843 34494502

[41] Tanasescu R Constantinescu CS Tench CR Manouchehrinia A. Smoking cessation and the reduction of disability progression in multiple sclerosis: a cohort study. Nicotine Tob Res. (2018) 20:589–95. 10.1093/ntr/ntx084 28402456

[42] Wergeland S Riise T Torkildsen O. Response to 'Seasonal variation of vitamin D and Epstein-Barr virus antibody in multiple sclerosis patients', a comment letter regarding 'Vitamin D, HLA-DRB1 and Epstein-Barr virus antibody levels in a prospective cohort of multiple sclerosis patients'. Eur J Neurol. (2018) 25:e104. 10.1111/ene.13719 30134049

[43] Thacker EL Mirzaei F Ascherio A. Infectious mononucleosis and risk for multiple sclerosis: a meta-analysis. Ann Neurol. (2006) 59:499–503. 10.1002/ana.20820 16502434

[44] Ascherio A Munger KL. Environmental risk factors for multiple sclerosis. Part I: the role of infection. Ann Neurol. (2007) 61:288–99. 10.1002/ana.21117 17444504

[45] Nielsen TR Rostgaard K Nielsen NM Koch-Henriksen N Haahr S Sorensen PS. Multiple sclerosis after infectious mononucleosis. Arch Neurol. (2007) 64:72–5. 10.1001/archneur.64.1.72 17210811

[46] Bach JF. The effect of infections on susceptibility to autoimmune and allergic diseases. N Engl J Med. (2002) 347:911–20. 10.1056/NEJMra020100 12239261

[47] Ascherio A Munch M. Epstein-Barr virus and multiple sclerosis. Epidemiology. (2000) 11:220–4. 10.1097/00001648-200003000-00023 11021623

[48] Alotaibi S Kennedy J Tellier R Stephens D Banwell B. Epstein-Barr virus in pediatric multiple sclerosis. JAMA. (2004) 291:1875–9. 10.1001/jama.291.15.1875 15100207

[49] Pohl D Krone B Rostasy K Kahler E Brunner E Lehnert M. High seroprevalence of Epstein-Barr virus in children with multiple sclerosis. Neurology. (2006) 67:2063–5. 10.1212/01.wnl.0000247665.94088.8d 17159123

[50] Ascherio A Munger KL Lennette ET Spiegelman D Hernan MA Olek MJ. Epstein-Barr virus antibodies and risk of multiple sclerosis: a prospective study. JAMA. (2001) 286:3083–8. 10.1001/jama.286.24.3083 11754673

[51] Sundstrom P Juto P Wadell G Hallmans G Svenningsson A Nystrom L. An altered immune response to Epstein-Barr virus in multiple sclerosis: a prospective study. Neurology. (2004) 62:2277–82. 10.1212/01.WNL.0000130496.51156.D7 15210894

[52] Levin LI Munger KL Rubertone MV Peck CA Lennette ET Spiegelman D. Temporal relationship between elevation of epstein-barr virus antibody titers and initial onset of neurological symptoms in multiple sclerosis. JAMA. (2005) 293:2496–500. 10.1001/jama.293.20.2496 15914750

[53] Delorenze GN Munger KL Lennette ET Orentreich N Vogelman JH Ascherio A. Epstein-Barr virus and multiple sclerosis: evidence of association from a prospective study with long-term follow-up. Arch Neurol. (2006) 63:839–44. 10.1001/archneur.63.6.noc50328 16606758

[54] Munger KL Levin LI O'Reilly EJ Falk KI Ascherio A. Anti-Epstein-Barr virus antibodies as serological markers of multiple sclerosis: a prospective study among United States military personnel. Mult Scler. (2011) 17:1185–1193. 10.1177/1352458511408991 21685232 PMC3179777

[55] Xu GJ Kula T Xu Q Li MZ Vernon SD Ndung'u T. Comprehensive serological profiling of human populations using a synthetic human virome. Science. (2015) 348:aaa0698. 10.1126/science.aaa0698 26045439 PMC4844011

[56] Kuchroo VK Weiner HL. How does Epstein-Barr virus trigger MS? Immunity. (2022) 55:390–2. 10.1016/j.immuni.2022.02.008 35263566

[57] Giovannoni G Hawkes CH Lechner-Scott J Levy M Yeh EA Gold J. Is EBV the cause of multiple sclerosis? Mult Scler Relat Disord. (2022) 58:103636. 10.1016/j.msard.2022.103636 35114510

[58] Moutschen M Leonard P Sokal EM Smets F Haumont M Mazzu P. Phase I/II studies to evaluate safety and immunogenicity of a recombinant gp350 Epstein-Barr virus vaccine in healthy adults. Vaccine. (2007) 25:4697–705. 10.1016/j.vaccine.2007.04.008 17485150

[59] Sokal EM Hoppenbrouwers K Vandermeulen C Moutschen M Leonard P Moreels A. Recombinant gp350 vaccine for infectious mononucleosis: a phase 2, randomized, double-blind, placebo-controlled trial to evaluate the safety, immunogenicity, and efficacy of an Epstein-Barr virus vaccine in healthy young adults. J Infect Dis. (2007) 196:1749–53. 10.1086/523813 18190254

[60] Elliott SL Suhrbier A Miles JJ Lawrence G Pye SJ Le TT. Phase I trial of a CD8+ T-cell peptide epitope-based vaccine for infectious mononucleosis. J Virol. (2008) 82:1448–57. 10.1128/JVI.01409-07 18032491 PMC2224445

[61] Kanekiyo M Bu W Joyce MG Meng G Whittle JR Baxa U. Rational design of an Epstein-Barr virus vaccine targeting the receptor-binding site. Cell. (2015) 162:1090–100. 10.1016/j.cell.2015.07.043 26279189 PMC4757492

[62] Bu W Joyce M. G. Nguyen H. Banh D. V. Aguilar F. Tariq Z.. (2019). Immunization with components of the viral fusion apparatus elicits antibodies that neutralize Epstein-Barr virus in b cells and epithelial cells. Immunity. 50:1305–1316.e1306. 10.1016/j.immuni.2019.03.010 30979688 PMC6660903

[63] Cui X Cao Z Ishikawa Y Cui S Imadome KI Snapper CM. Immunization with Epstein-Barr virus core fusion machinery envelope proteins elicit high titers of neutralizing activities and protect humanized mice from lethal dose EBV challenge. Vaccines (Basel). (2021) 9. 10.3390/vaccines9030285 33808755 PMC8003492

[64] Escalante GM Foley J Mutsvunguma LZ Rodriguez E Mulama DH Muniraju M. A pentavalent epstein-barr virus-like particle vaccine elicits high titers of neutralizing antibodies against Epstein-Barr virus infection in immunized rabbits. Vaccines (Basel). (2020) 8169. 10.3390/vaccines8020169 32268575 PMC7349562

[65] Ruiss R Jochum S Wanner G Reisbach G Hammerschmidt W Zeidler R. A virus-like particle-based Epstein-Barr virus vaccine. J Virol. (2011) 85:13105–13. 10.1128/JVI.05598-11 21994444 PMC3233152

[66] NCT05164094. A Study of an Epstein-Barr Virus (EBV) Candidate Vaccine, mRNA-1189, in 18- to 30-Year-Old Healthy Adults. Available onine at: https://clinicaltrials.gov/ct2/show/NCT05164094 [Accessed].

[67] NCT04645147. Safety and Immunogenicity of an Epstein-Barr Virus (EBV) gp350-Ferritin Nanoparticle Vaccine in Healthy Adults With or Without EBV Infection [Online]. Available online at: https://clinicaltrials.gov/ct2/show/NCT04645147 [Accessed].

[68] Brooks JM Long HM Tierney RJ Shannon-Lowe C Leese AM Fitzpatrick M. Early T cell recognition of B cells following epstein-barr virus infection: identifying potential targets for prophylactic vaccination. PLoS Pathog. (2016) 12:e1005549. 10.1371/journal.ppat.1005549 27096949 PMC4838210

[69] Dowell AC Haigh TA Ryan GB Turner JE Long HM Taylor GS. Cytotoxic CD4+ T-cells specific for EBV capsid antigen BORF1 are maintained in long-term latently infected healthy donors. PLoS Pathog. (2021) 17:e1010137. 10.1371/journal.ppat.1010137 34882759 PMC8691624

[70] Lin CL Lo WF Lee TH Ren Y Hwang SL Cheng YF. Immunization with Epstein-Barr Virus (EBV) peptide-pulsed dendritic cells induces functional CD8+ T-cell immunity and may lead to tumor regression in patients with EBV-positive nasopharyngeal carcinoma. Cancer Res. (2002) 62:6952–8. 12460912

[71] Li J Chen QY He J Li ZL Tang XF Chen SP. Phase I trial of adoptively transferred tumor-infiltrating lymphocyte immunotherapy following concurrent chemoradiotherapy in patients with locoregionally advanced nasopharyngeal carcinoma. Oncoimmunology. (2015) 4:e976507. 10.4161/23723556.2014.976507 25949875 PMC4404907

[72] Chia WK Wang WW Teo M Tai WM Lim WT Tan EH. A phase II study evaluating the safety and efficacy of an adenovirus-DeltaLMP1-LMP2 transduced dendritic cell vaccine in patients with advanced metastatic nasopharyngeal carcinoma. Ann Oncol. (2012) 23:997–1005. 10.1093/annonc/mdr341 21821548 PMC3314324

[73] Si Y Deng Z Lan G Du H Wang Y Si J. The Safety and Immunological Effects of rAd5-EBV-LMP2 vaccine in nasopharyngeal carcinoma patients: a phase I clinical trial and two-year follow-up. Chem Pharm Bull (Tokyo). (2016) 64:1118–23. 10.1248/cpb.c16-00114 27477649

[74] Hui EP Taylor GS Jia H Ma BB Chan SL Ho R. Phase I trial of recombinant modified vaccinia ankara encoding Epstein-Barr viral tumor antigens in nasopharyngeal carcinoma patients. Cancer Res. (2013) 73:1676–88. 10.1158/0008-5472.CAN-12-2448 23348421 PMC6485495

[75] Taylor GS Jia H Harrington K Lee LW Turner J Ladell K. A recombinant modified vaccinia ankara vaccine encoding Epstein-Barr Virus (EBV) target antigens: a phase I trial in UK patients with EBV-positive cancer. Clin Cancer Res. (2014) 20:5009–22. 10.1158/1078-0432.CCR-14-1122-T 25124688 PMC4340506

[76] Robinson WH Steinman L. Epstein-Barr virus and multiple sclerosis. Science. (2022) 375:264–5. 10.1126/science.abm7930 35025606

[77] Nelson P Rylance P Roden D Trela M Tugnet N. Viruses as potential pathogenic agents in systemic lupus erythematosus. Lupus. (2014) 23:596–605. 10.1177/0961203314531637 24763543

[78] Bakkalci D Jia Y Winter JR Lewis JE Taylor GS Stagg HR. Risk factors for Epstein Barr virus-associated cancers: a systematic review, critical appraisal, and mapping of the epidemiological evidence. J Glob Health. (2020) 10:010405. 10.7189/jogh.10.010405 32257153 PMC7125417

[79] Van Zyl DG Mautner J Delecluse HJ. Progress in EBV Vaccines. Front Oncol. (2019) 9:104. 10.3389/fonc.2019.00104 30859093 PMC6398348

[80] Ruhl J Leung CS Munz C. Vaccination against the Epstein-Barr virus. Cell Mol Life Sci. (2020) 77:4315–24. 10.1007/s00018-020-03538-3 32367191 PMC7223886

[81] Handel AE Giovannoni G Ebers GC Ramagopalan SV. Environmental factors and their timing in adult-onset multiple sclerosis. Nat Rev Neurol. (2010) 6:156–66. 10.1038/nrneurol.2010.1 20157307

[82] Levin LI Munger KL O'Reilly EJ Falk KI Ascherio A. Primary infection with the Epstein-Barr virus and risk of multiple sclerosis. Ann Neurol. (2010) 67:824–830. 10.1002/ana.21978 20517945 PMC3089959

[83] Hedstrom AK Huang J Michel A Butt J Brenner N Hillert J. High levels of Epstein-Barr virus nuclear antigen-1-specific antibodies and infectious mononucleosis act both independently and synergistically to increase multiple sclerosis risk. Front Neurol. (2019) 10:1368. 10.3389/fneur.2019.01368 32038456 PMC6992610

[84] Zdimerova H Murer A Engelmann C Raykova A Deng Y Gujer C. Attenuated immune control of Epstein-Barr virus in humanized mice is associated with the multiple sclerosis risk factor HLA-DR15. Eur J Immunol. (2021) 51:64–75. 10.1002/eji.202048655 32949466

[85] Cata-Preta BO Santos TM Mengistu T Hogan DR Barros AJD Victora CG. Zero-dose children and the immunisation cascade: understanding immunisation pathways in low and middle-income countries. Vaccine. (2021) 39:4564–70. 10.1016/j.vaccine.2021.02.072 33744046 PMC8314014

[86] Kuri A Jacobs BM Vickaryous N Pakpoor J Middeldorp J Giovannoni G. Epidemiology of Epstein-Barr virus infection and infectious mononucleosis in the United Kingdom. BMC Public Health. (2020) 20:912. 10.1186/s12889-020-09049-x 32532296 PMC7291753

[87] Power Analysis Sample Size Software. Tests for Two Survival Curves Using Cox's Proportional Hazards Mode. Kaysville, UT: NCSS; LLC (2021). Available online at: http:ncss.com/software/pass

[88] Lunemann JD Edwards N Muraro PA Hayashi S Cohen JI Munz C. Increased frequency and broadened specificity of latent EBV nuclear antigen-1-specific T cells in multiple sclerosis. Brain. (2006) 129:1493–506. 10.1093/brain/awl067 16569670

[89] Ruprecht K Wunderlich B Giess R Meyer P Loebel M Lenz K. Multiple sclerosis: the elevated antibody response to Epstein-Barr virus primarily targets, but is not confined to, the glycine-alanine repeat of Epstein-Barr nuclear antigen-1. J Neuroimmunol. (2014) 272:56–61. 10.1016/j.jneuroim.2014.04.005 24798244

[90] Cencioni MT Magliozzi R Nicholas R Ali R Malik O Reynolds R. Programmed death 1 is highly expressed on CD8(+) CD57(+) T cells in patients with stable multiple sclerosis and inhibits their cytotoxic response to Epstein-Barr virus. Immunology. (2017) 152:660–76. 10.1111/imm.12808 28767147 PMC5680058

[91] Pender MP Csurhes PA Burrows JM Burrows SR. Defective T-cell control of Epstein-Barr virus infection in multiple sclerosis. Clin Transl Immunology. (2017) 6:e126. 10.1038/cti.2016.87 28197337 PMC5292561

[92] Pender MP Csurhes PA Smith C Douglas NL Neller MA Matthews KK. Epstein-Barr virus-specific T cell therapy for progressive multiple sclerosis. JCI Insight. (2018) 3:e124714. 10.1172/jci.insight.124714 30429369 PMC6302936

[93] Ioannides ZA Csurhes PA Douglas NL Mackenroth G Swayne A Thompson KM. Sustained clinical improvement in a subset of patients with progressive multiple sclerosis treated with epstein-barr virus-specific T cell therapy. Front Neurol. (2021) 12:652811. 10.3389/fneur.2021.652811 33790852 PMC8005645

